# Conditional-GAN Based Data Augmentation for Deep Learning Task Classifier Improvement Using fNIRS Data

**DOI:** 10.3389/fdata.2021.659146

**Published:** 2021-07-29

**Authors:** Sajila D. Wickramaratne, Md.Shaad Mahmud

**Affiliations:** Department of Electrical and Computer Engineering, University of New Hampshire, Durham, NH, United States

**Keywords:** functional near-infrared spectroscopy, deep learning, classification, GAN, CGAN, CNN

## Abstract

Functional near-infrared spectroscopy (fNIRS) is a neuroimaging technique used for mapping the functioning human cortex. fNIRS can be widely used in population studies due to the technology’s economic, non-invasive, and portable nature. fNIRS can be used for task classification, a crucial part of functioning with Brain-Computer Interfaces (BCIs). fNIRS data are multidimensional and complex, making them ideal for deep learning algorithms for classification. Deep Learning classifiers typically need a large amount of data to be appropriately trained without over-fitting. Generative networks can be used in such cases where a substantial amount of data is required. Still, the collection is complex due to various constraints. Conditional Generative Adversarial Networks (CGAN) can generate artificial samples of a specific category to improve the accuracy of the deep learning classifier when the sample size is insufficient. The proposed system uses a CGAN with a CNN classifier to enhance the accuracy through data augmentation. The system can determine whether the subject’s task is a Left Finger Tap, Right Finger Tap, or Foot Tap based on the fNIRS data patterns. The authors obtained a task classification accuracy of 96.67% for the CGAN-CNN combination.

## 1 Introduction

Functional near-infrared spectroscopy (fNIRS) is a neuroimaging technology for mapping the functioning of the human cortex, which exploits near-infrared spectroscopy (Ferrari and Quaresima, 2012). This mapping is done by measurements and images of local brain changes caused by the modulation of cerebral blood flow and oxygen metabolism by neural activity (Yücel et al., 2017). fNIRS is a non-invasive, repeatable, portable, high temporal resolution, and economical technology with widespread use. Advances in technology and hardware have allowed fNIRS researchers to non-invasively probe neurovascular physiology, increasing resolution and signal quality (Hocke et al., 2018). However, technologies such as EEG have the limitations of imprecise localization and inaccessibility of sub-cortical areas (Naseer and Hong, 2015). Other techniques such as fMRI can be used to measure hemodynamic activities yet, due to cost and portability, may not be suitable for population studies. Further, fNIRS has a better temporal resolution than fMRI in most cases (Huppert et al., 2006). fNIRS is more suited for the population studies for which other imaging modalities are of limited use. Such studies can include infants and children, procedures involving mobility and interactivity, and clinical environments (Yücel et al., 2017).

With the popularity of brain-computer interfaces, task classification using neural imaging technologies has become more critical. When it comes to neuroimaging techniques used for BCI, both EEG and fNIRS have emerged to be the most widespread for task classification (Hong et al., 2018; Saadati et al., 2019; Shin et al., 2016). Although there are much more sophisticated and accurate neuroimaging techniques that can be used for medical diagnosis, EEG and fNIRS are much suitable in studies with population since it is inexpensive and not harmful to repeated use. Most of the existing task classification systems use conventional machine learning methods for classification. The traditional machine learning methods are used frequently due to their simplicity of implementation. On the drawbacks, these traditional methods require a significant amount of data preprocessing and a feature extraction phase.

Additionally, traditional machine learning methods may not capture all the valuable information in complex neural signal patterns. The accuracy of the conventional classifiers depends predominantly on the features selected for training the model. The extraction and selection of optimal features can be a challenge with neural signals. The complexity and multi-dimensionality of neuroimaging data make it much more suitable for deep learning methods.

There are currently some successful deep learning classifiers with neuroimaging modalities such as EEG and fNIRS (Hennrich et al., 2015; Chiarelli et al., 2018). One of the main issues with implementing deep learning-based classifiers is the sample size. The models tend to overfit with small sample sizes, create difficulty generalizing the model, and underperform testing data. Data augmentation is a method that enables researchers to increase the diversity of training data available for models without additional data collection. In the health field, obtaining high-quality labeled data for deep learning algorithms can be costly and time-consuming. This is a situation where generative networks can be helpful. Traditional data augmentation methods include operations such as zooming, cropping and rotating, etc. These methods can be very successful in object classification. In cases where there is no single object to focus on classification, such time-series data represented in images, using traditional data augmentation methods makes little sense. The traditional data augmentation methods may not be suitable for medical data generation, where a strict format is adhered to on many occasions. One way which can be used for data augmentation with a deep learning algorithm is known as General Adversarial Networks (GAN). Researchers have found that data augmentation with GAN networks has improved the classification accuracy (Antoniou et al., 2017).

The authors propose a classification system based on a hybrid CGAN-CNN network to classify images derived from fNIRS signals. The system can determine whether the task performed by the subject is a Left Finger Tap, Right Finger Tap, or Foot Tap. The proposed deep learning system will not be affected by the relatively smaller number of samples due to the ability of the CGANs to augment the data. This proposed can be used when training a deep learning classifier with a relatively smaller number of samples. The proposed system can both generate artificial samples and classify real data as well. The proposed system obtained a classification accuracy of 96.67% and an average AUROC of 0.98. This proposed system exceeded the accuracy obtained for the same dataset using the traditional machine learning classifiers.

## 2 Background

**FIGURE 1:**
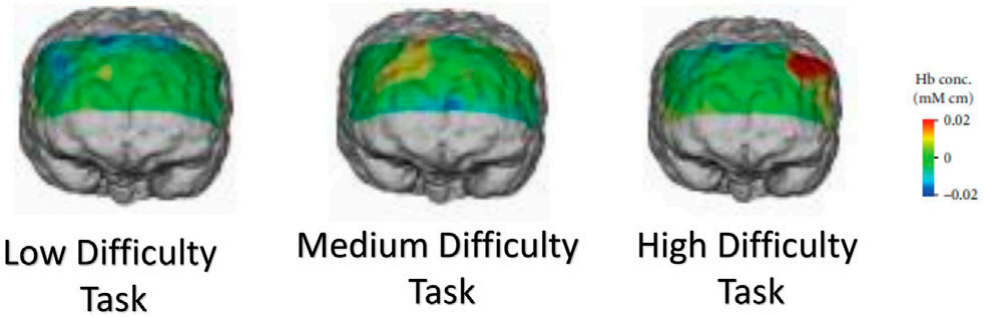
Functional brain imaging by fNIRS obtained while a subject was performing low, medium and high difficulty tasks during driving (Tsunashima and Yanagisawa, 2009).

fNIRS use near-infrared rays to measure changes in cerebral blood flow non-invasively. Its measurement principle is based on the measurement of hemoglobin’s oxygenation in the cerebral blood flow (Jobsis, 1977). fNIRS are actively used to classify tasks in brain-computer interfaces. Due to its economic and portable nature, it is widely used for population studies. Researchers can visualize the cerebral blood flow using fNIRS signals to analyze how different cortex parts are activated during certain tasks. As an example, Figure 1 illustrates how brain activation patterns can be visualized using fNIRS brain imaging when the subject is performing tasks of varying intensity while driving (Tsunashima and Yanagisawa, 2009). fNIRS data can be used to classify a wide variety of tasks that include cognitive tasks such as mental arithmetic and motor imagery tasks such as finger/foot tapping (Shin et al., 2018; Bak et al., 2019).

Acquiring medical data present practical difficulties due to time, money, labor, and economic cost. The deep learning-based model can better perform medical image classification than hand-crafted features when dealing with a large amount of data (Zhang et al., 2019). Artificial data can be generated by using traditional image augmentation methods. However, the images generated by traditional augmentation methods have a similar distribution to the original. This practice may not be suitable when the artificial samples have to represent data distribution among different subjects. GANs provide a method to augment the training data with artificially generated samples. GANs have successfully performed in many areas, including image and vision computing to speech and language processing (Wang et al., 2017). GANs have been used in the medical field where synthetic image generation has improved the classification accuracy with CNN networks where collecting an extensive amount of data is not feasible (Frid-Adar et al., 2018).

GANs are an innovative way of training a generative model by framing the problem as a supervised learning problem using deep learning models. GANs can automatically discover the pattern in input data. The GAN architecture was first proposed in the 2014 paper by Goodfellow et al. (2014). GANs can generate new samples that appear to belong to the original dataset (Hong et al., 2019).

There are two sub-models in a GAN called the Generator (G) and Discriminator (D). The generator model is used to train to generate new examples and the discriminator model to classify samples real or fake generated (Creswell et al., 2018). GANs are the two models behind the training motivation trying to achieve the Nash equilibrium of Game Theory (Nash, 1951). A non-cooperative game solution must be reached between two adversaries to achieve Nash equilibrium. Each player already knows all the other player’s strategies. Therefore, no player gains anything by modifying their strategy (Goodfellow et al., 2014). Any function that can be differentiated can be used as the function for equations of Generator and Discriminator.

The generator model takes a fixed-length random vector as input and generates a sample in the domain. From a Gaussian distribution, a random vector is drawn to initiate the generative process. After training, points in this multidimensional vector space will correspond to points in the problem domain to form a compressed representation of the data distribution. This vector space is known as a latent space. Latent variables though necessary for the domain, are not directly observable.

In GANs, when the Discriminator successfully identifies real and fake samples, it is rewarded, or no change is needed to the model parameters (Goodfellow et al., 2014). In contrast, the Generator is penalized with extensive updates to model parameters. This process is analogous to a zero-sum game. When the optimum solution is reached, the Generator can generate perfect duplicates from the input domain every time. In this case, the Discriminator cannot differentiate between real and fake samples and predict their authenticity in every case.

An essential extension to the GAN is in their use of conditionally generating an output (Mirza and Osindero, 2014). The generative model can be trained to create new examples from the input domain. Some additional input conditions the random vector from the latent space. In a conditioned Discriminator, additional input is given along with the input images. In the classification label type conditional input, the Discriminator would expect the class’s input. The Generator is taught to generate examples of that class to fool the Discriminator. In this way, a conditional GAN can generate samples from a domain of a given type.

One of the many significant advancements in using deep learning methods in computer vision domains is data augmentation to improve model performance. GANs have increasingly been used for data augmentation (Douzas and Bacao, 2018). Further augmentation can increase model skill, provide a regularizing effect, and reduce generalization error. It works by creating artificial but plausible examples from the input problem domain on which the model is trained. Traditional augmentation methods consist of simple transforms of existing images such as crops, flips, etc. Generative models, if trained successfully, can provide a more domain-specific approach to data augmentation.

## 3 Materials and Methods

### 3.1 Overall System

**FIGURE 2:**
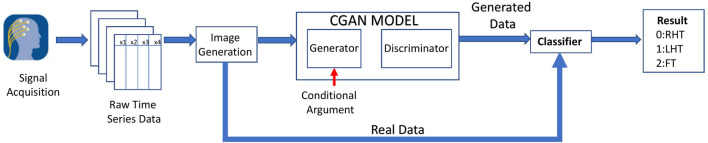
Overall architecture of the proposed system.

Several analyzes were performed on the fNIRS to determine the final configuration of the system design. Figure 2 illustrates the Overview of the complete system. The acquired raw fNIRS data were initially preprocessed to remove the disturbances. Neural signals have highly correlated variables that should be removed before being fed into the model. Therefore dimension reduction techniques were performed on preprocessed data to remove the highly correlated variables. Afterward, the data is sent to the image generation phase. The Grammian Angular Summation Field images are generated for the time series. The data set is then divided into the test and training sets. The training set is sent to the CGAN model, where artificial images are generated for the three categories. Both real data and CGAN generated data are used to train the deep learning classifier based on CNN. The test set is fed directly to the classifier and used to determine the performance. For the baseline classifiers that used features, a separate feature extraction process was performed, briefly described in later sections. Finally, the result is obtained with the task being classified into Right Hand Tap (RHT), Left Hand Tap (LHT), or Foot Tap (FT).

### 3.2 Data

**FIGURE 3:**
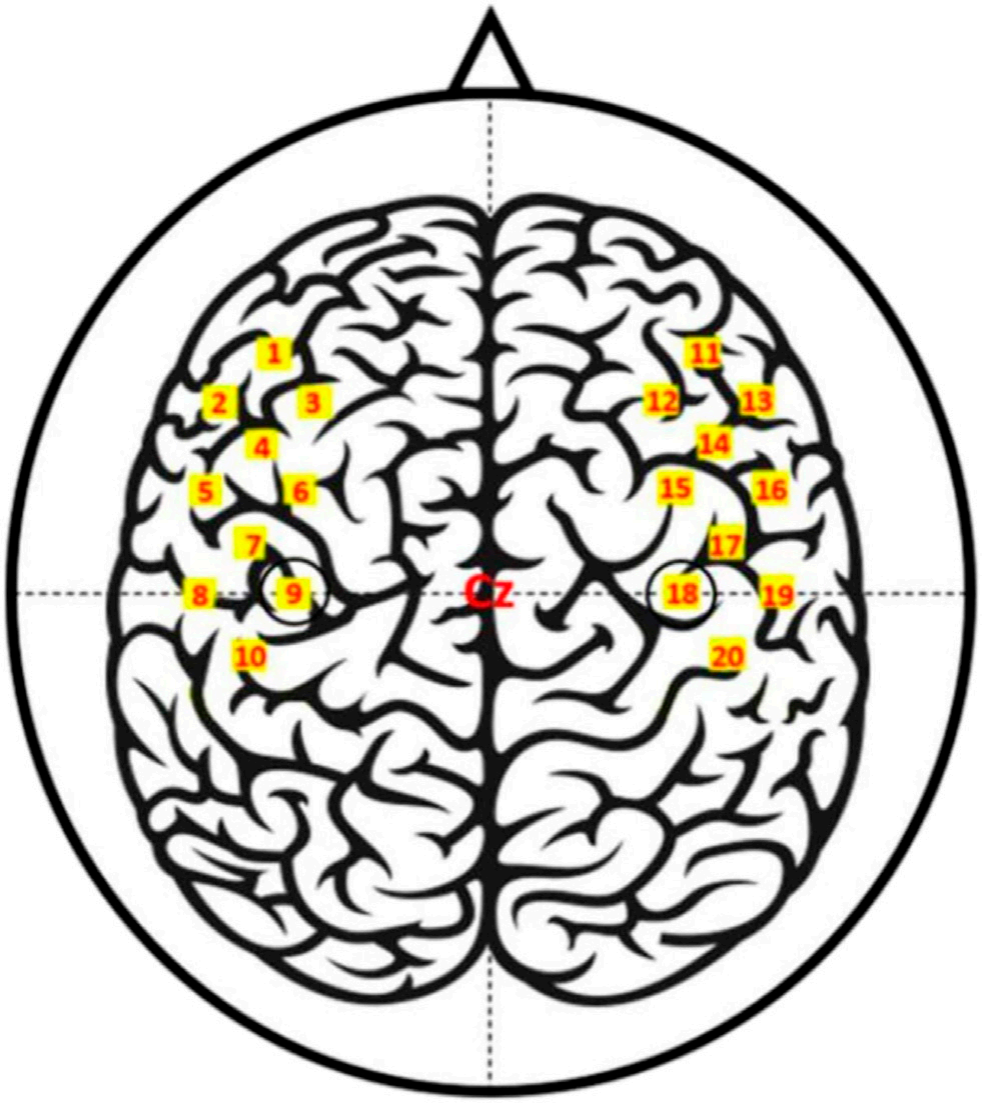
fNIRS channel locations (Bak et al., 2019).

The data used for the training of the classifier was obtained from an open database (Bak et al., 2019). A more detailed description of the data can be found in the original publication. Thirty volunteers participated in this study. A total of 30 volunteers without a history of psychiatric or neurological disorders participated in the experiment (17 males 13 females; 23.4 ± 2.5 years old) (Bak et al., 2019). The fNIRS data were recorded by a multichannel fNIRS system consisting of eight light sources and eight detectors. Figure 3 illustrates the placement of the fNIRS optodes. A single trial included an introduction period and a task period, followed by an inter-trial break. The inter-trial interval was 30 s on average. Out of RHT, LHT, and FT, a specific task type was displayed randomly, which volunteers were required to perform. For RHT/LHT tasks, the volunteers performed unilateral complex finger-tapping at a rate of 2 Hz. For FT, the participants tapped their foot at a 1 Hz rate.

**FIGURE 4:**
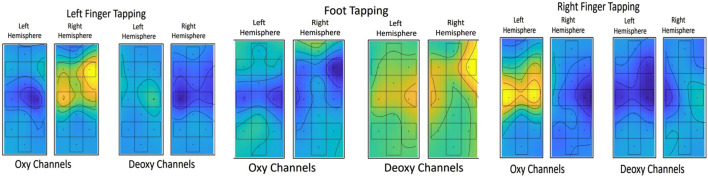
Brain Imaging Representation (Oxy and Deoxy channels) for the three tasks.

fNIRS is a brain imaging technique used to observe the local changes in hemoglobin concentrations in the brain that arise from cerebral blood flow modulation. Certain brain areas are activated when engaged in a task, thus changing the oxy and deoxy patterns. Figure 4 shows the topographic distributions plotted using oxy and deoxy channels. From the topographic images, it is evident that the left hemisphere responds with an increase in HbO to the right finger-tapping task and the right hemisphere to the left finger-tapping task; the HbR shows the opposite pattern. Interestingly, both the left and right hemispheres show decreased HbO and an increase in HbR during the tapping foot.

### 3.3 Pre-Processing

The fNIRS signals can contain various disturbances such as instrumental, experimental, and physiological noises. Instrumental and experimental noises are usually removed before converting the raw optical density signals to the concentration changes of HbO and HbR(Naseer and Hong, 2015). The physiological noises have to be removed from the HbO and HbR changes. Physiological noises consist of heartbeat (1–1.5 Hz), respiration (0.2–0.5 Hz), Mayer waves (0.1 Hz), among others. After experimenting with several filtering arrangements, the filtering method recommended by the original authors who published the dataset was used. None of the filtering methods showed a significant advantage over the other. A zero-order filter implemented by the third-order Butterworth filter with a 0.01–0.1 Hz passband was used to remove the physiological noises and DC offset for this dataset (Bak et al., 2019). The ΔHbO/R values were segmented into epochs ranging from −2–28 s relative to the task onset. Baseline correction was done for each epoch by subtracting the average value within the reference interval (−1–0 s).

The ΔHbO/R features were extracted from three-time windows in the ranges of 0–5, 5–10 and 10–15 s. Epochs were employed to compute the average ΔHbO/R for each of the 20 channels. Since no feature/channel selection method was applied, the feature vector comprised three features extracted from 20 channels. The feature vector’s dimensionality was computed as 120. Before being fed to the model, the feature vectors were standardized.

EEG and NIRS data have high dimensionality, with multiple variables which are highly correlated. This may cause poor performance of the machine learning algorithms. Hence it is essential to use methods such as Independent Component Analysis (ICA), or Principal Component Analysis (PCA) can be used dimensionality reduction and maximize the statistical independence of the estimated components (Comon, 1994). In this study, kernel PCA, an extension to the traditional PCA technique with the ability to extract principal nonlinear components without expensive computations, is used instead of PCA due to the nonlinear nature of data (Mika et al., 1999). Hence, before preparing the data for the deep learning classifier Kernal, PCA was applied to remove highly correlated variables.

#### 3.3.1 Image Generation

Image generation is a critical part of a classification system based on CNN. In the proposed method, CNN’s are used for both generation and classification phases. The quality of the images fed to these networks will determine the performance of the classifiers. In this instance, fNIRS signals pose a challenge to CNN-based systems since they can be represented differently. This section will discuss the different images used and how they finally decide which category of images to train the classifier. Recently, remarkable results have been achieved by processing data with deep learning techniques and, specifically, by using GAN networks with images as input. GAN networks are typically associated with the neural network’s outstanding performance for reading, processing, and extracting two-dimensional data’s essential features, which have positively contributed to its popularity. However, even in scenarios where input data aren’t formatted as an image, many transformation methods have helped apply CNNs to other data types. The time series is one of these data structures modeled to approach a computer vision perspective.

The time-series data must be converted to a 2D image for input to the CNN. A temporal approach is typically chosen in previous studies for this step, capturing all fNIRS channels in a time window. This method’s success depends heavily on the length of the dataset and the time window; a small number of samples could be overfitting. Only a few techniques can be used to represent the whole time series in its entirety. This section describes some possible solutions considered in this study to determine which image generation process was suitable for the classification system.

In Spectrograms, time series carry information with both time and frequency as magnitude dimensions. Local relationships are represented using different domain spectrograms. This feature complicates the local feature extraction feeding two-dimensional CNN layers with spectrograms, as they have non-local relationships. Several CNN-based classification systems have used spectrograms of neural signals (Ho et al., 2019; Chhabra et al., 2020).

A Gramian Angular Field (GAF) is an image obtained from a 1-dimensional time series, representing some temporal correlation between each time point (Wang and Oates, 2015a). GAFs can be either Gramian Angular Summation Field (GASF) or Gramian Angular Difference Field (Wang and Oates, 2015b). A GAF, in which we represent time series in a polar coordinate system instead of the typical Cartesian coordinates. For this study, GASF images were used as the images to train the Convolution Neural Networks. After analyzing the best-performing baseline classifier’s feature importance, the fNIRS channel used to generate the images was chosen. GAFs have been used to classify neural signals such as EEG waveforms and fNIRS (Thanaraj et al., 2020; Wickramaratne and Mahmud, 2021).Another method used to convert a time series to an image is a Recurrence plot, an image obtained from a time series representing the distances between each time point (Marwan et al., 2007). For multivariate time series, a joint recurrence plot derived from the individual recurrence plot can be used. Researchers have used recurrence Plots to classify EEG signals primarily for medical conditions (Zeng et al., 2020; Gao et al., 2020). After initial analysis, Grammian Summation Fields were chosen for the proposed classification system. In the first step of image generation, the time series are analyzed. From the fNIRS stream, the area where the tasks are performed is isolated. A single recording contains additional data in addition to the task completed. This data is not required for the classification and can work as noise in the system. Afterward, the chosen data stream contains many highly correlated channels, is sent through the kernel PCA. After the initial image is generated, the images are preprocessed by rescaling them according to CNN’s dimensions. Also, the images are grey-scaled to make sure the pixels’ values are within a range.

### 3.4 Model

The proposed system consists of two models—one system for classifying samples and the Generative model to generate synthetic samples. Since the inputs used in the classification system are image data and a relatively small number of samples are available for the model’s training, a generative adversarial network-based approach is used. In this specific case, a Conditional GAN network was used to generate synthetic samples used to train the model. The GAN network has two competing models known as the Generator and Discriminator. The structure of these individual models is detailed in later sections. One of the most challenging problems in the GAN network is training the models in an adversarial manner. Stable training mechanism for training GANs where both models can attain a status equivalent to Nash Equilibrium.

#### 3.4.1 Model Architecture

After experimenting with several generative models, a conditional GAN network was chosen to create the synthetic samples. A CGAN network has the unique ability to generate new samples of a given category by passing a conditional argument to the Generator. The Generator will be generating the synthetic samples according to the conditional argument.

GANs are predominantly associated with the image data and use Convolutional Neural Networks (CNNs) as the generator and discriminator models. Remarkable progress has been seen using CNNs more generally to achieve state-of-the-art results on a suite of computer vision tasks. The Generator’s input provides a compressed representation of the set of images utilized for model training. The Generator generates new images, which can be easily viewed and assessed by the developers. GANs gives the ability to determine the quality of the generated images visually. This unique feature and the advances in the computer vision field have made GANs the most sought-after generative model.

Following is the mathematical representation of loss function (or objective function) of GANs, as shown in Eq. 1.

$$\underset{G}{min}\;\underset{D}{max}V\left(G,D\right)=\underset{G}{min}\;\underset{D}{max}\;E_{x∼p_{data}}\left[logD\left(x\right)\right]+E_{z∼p_{z}}\left[log\left(1-D\left(G\left(z\right)\right)\right)\right]$$ (1)

where *x* is a sample from the real dataset distribution *pdata*(*x*) and *z* is sampled from a latent space distribution *pz*(*z*). Eq. 1 shows the two networks playing a Mini-Max Game, each trying to improve their loss function.

The technique of CGAN is very similar to GAN. Both the Generator and Discriminator have conditioned an extra input(y). This conditioning can be performed by feeding into both the Discriminator and Generator as the additional input layer. “Y” can be any auxiliary information. In the proposed model, class labels are considered as “y” parameter (Gauthier, 2014). The cost function for CGAN is shown in Eq. 2. The intuition behind the conditional information, y, is that by adding additional information, both the generator G and the discriminator D learn to operate in specific modes.

$$\underset{G}{min}\;\underset{D}{max}\;V\left(G,D\right)=\underset{G}{min}\;\underset{D}{max}\;E_{x∼p_{data}}\left[logD\left(x|y\right)\right]+E_{z∼p_{z}}\left[log\left(1-D\left(G\left(z|y\right)\right)\right)\right]$$ (2)

#### 3.4.2 Classifier

**FIGURE 5:**
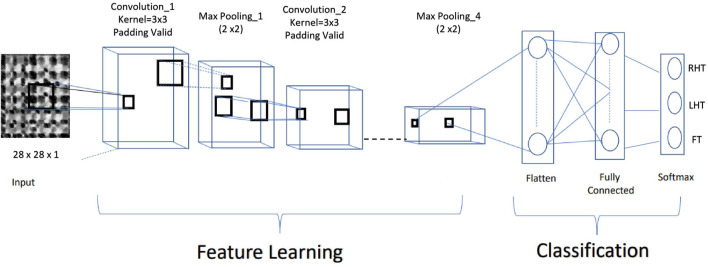
CNN based architecture of the proposed classifier.

The classifier used to determine the participant’s task was based on CNN architecture, illustrated in Figure 5 The model uses both real data and synthetic data generated by the GAN network. The network was tested only on real data. CNN uses a multidimensional structure in which each set of neurons explores a small region of the image. Each group of neurons specializes in identifying one part of the image. The final output is a vector of probability scores, representing how likely each feature is to be part of a class.

A CNN operates in three stages. The first is a convolution. The image is scanned a few pixels at a time, and a feature map is created with probabilities that each feature belongs to the required class. The second stage is pooling or down sampling, which reduces each feature’s dimensionality while maintaining its most relevant information. The pooling stage creates an overview of the essential features in the image. Max Pooling is commonly used in CNNs, in which the highest value is taken from each pixel area scanned by the CNN. Usually, CNN has to perform several rounds of convolution and pooling. CNNs can search for the appropriate features by themselves. Hence an additional feature selection step is not required.

This fully connected neural network analyzes the final probabilities and decides to which class the image belongs. The fully connected layers perform classification on the extracted features based on information on labeled training data. Every node in a fully connected layer is connected to every node in the previous layer. Finally, the output layer contains a single node for each target class in the model with a softmax activation function to compute each class’s probability. The softmax activation function ensures that the final outputs fulfill the constraints of a probability density.

As shown in Figure 5, the CNN model consisted of 18 layers, including an input layer, four pairs of convolutional, max-pooling layers, Batch Normalization, two fully connected layers, and finally a softmax layer to obtain the classified class. All the Convolution Layers were activated by the Rectified Linear Unit (Relu) function. The proposed CNN classifier inputs fixed-size grey-scaled images of 28 × 28, with an intensity range rescaled (0,1).

A Dropout factor is applied to all hidden layers’ outputs, and all layers have an l2-kernel regularizer of strength 0.5 initially (Cogswell et al., 2015). The model was trained using batches of four and included images generated from the Generator in addition to real data. The learning rate was reduced on the plateau, and early stopping was used to reduce over-fitting. The loss function used was categorical cross-entropy for the model, and the optimizer was RMSprop (Tieleman and Hinton, 2012).

The hyper-parameters for the CNN network were chosen after a random search (Bergstra and Bengio, 2012). Hyperopt Python library was used for the random search (Bergstra et al., 2013). The chosen hyperparameters included dropout value, choice of the optimizer, kernel size, no. of neurons. The hyper-parameters which were able to achieve the best accuracy were chosen.

#### 3.4.3 Generator

The GAN network consists of two separate models: the Generator (G) and the Discriminator (D). G is used for producing fake samples similar to real data space from the latent variable z. D determines whether its input comes from G or real data space. G and D compete to achieve their individual goals; hence the term “adversarial” is used. Since D wants to classify real or fake samples, *V* (*G*, *D*) is considered an objective function as an aspect of the classification problem. From D’s perspective, if a sample comes from real data, D will maximize its output. In contrast, if a sample comes from G, D will minimize its output. Hence log (1 − *D* (*G* (*z*|*y*))) term appears in Eq. 1. Since G’s objective is to deceive D, it tries to maximize D’s output when a fake sample is presented to D. Consequently, D tries to maximize *V* (*G*, *D*). In contrast, G tries to minimize *V* (*G*, *D*), thus forming the minimax relationship in Eq. 1. In theory, when the equilibrium between G and D occurs when *pdata*(*x*) = *pg*(*x*) and *D* always produce 1/2, where *pg*(*x*) means a probability distribution of the data provided by the Generator.

The Generator takes a point in latent space and a class label as input. The output of the Generator is a grayscaled image of size 28 × 28 × 1 of a Gramian Angular Summation Field. The network architecture consists of a fully connected layer reshaped to size 7 × 7 × 128 and three deconvolutional layers to up-sample the image with a 4 × 4 kernel size. Deconvolution can be considered as expanding the pixels by inserting zeros in between them (Frid-Adar et al., 2018). Convolution over the expanded image will result in a larger output image. Batch normalization (BN) is applied to each layer except the output layer. BN helps to stabilize learning and issues with parameters’ initialization. This is useful to prevent models from falling into mode collapse. When mode collapse happens, the Generator will output same looking images with little diversity for different inputs. ReLU activation functions are applied to all layers except the output layer, where the tanh activation function is used. The Discriminator’s feedback helps the Generator to adjust its weights towards better performance.

#### 3.4.4 Discriminator

The Discriminator network is used to determine whether the generated samples can be considered as real samples. Typically the Discriminator can be regarded as working optimally to classify 50% of the generated sample as fake. GAN networks are formulated in two steps where the Discriminator is trained to maximize. The optimal Discriminator has the shape given in Eq. 3. The discriminator network has a typical CNN architecture that takes the input image of size 28 × 28 × 1 and outputs whether the image is real or fake.

$$D*\left(x\right)=\frac{P_{r}\left(x\right)}{P_{r}\left(x\right)+P_{g}\left(x\right)}$$ (3)

#### 3.4.5 Model Training

The training of GAN networks is one of the most challenging tasks. The generative models’ goal is to match the real data distribution *pdata*(*x*) from *pg*(*x*). Thus, minimizing differences between the two distributions is a crucial point for training generative models. Two competing systems should be trained at the same time. The training of the two systems is a zero-sum problem. The optimum solution can only be attained when the Nash Equilibrium is reached.

G and D are two differentiable functions that represent the Generator and the Discriminator, respectively. Inputs given to D are x (real data) and z (random data). G’s output is fake data produced per the probability distribution of actual data (or *pdata*), G (z). If existing data are given as input to Discriminator, it should classify the input data as real data, labeling it 1. Suppose fake or generated data is provided as input to Discriminator. In that case, it should classify the input data as fake data, labeling it 0. Discriminator strives to classify the input data correctly as per the source of data. The Generator seeks to deceive the Discriminator by making generated data G (z) similar and in line with the real data x. This game-like adversarial process improves the performance of both Discriminator and Generator slowly and gradually throughout the process. Therefore, slowly, Generator can generate better images that look more real because it has to fool the improved and more efficient Discriminator.

Although the GANs are increasing in popularity, they remain challenging to train, with most researchers finding stable architectures heuristically (Radford et al., 2015). Traditional approaches to generative modeling rely on maximizing likelihood or equivalently minimizes the Kullback-Leibler (KL) divergence between the original data distribution *P*_*r*_ and the Generator’s distribution *P*_*g*_ Notably, the discriminator model’s performance is used to update both the Discriminator model’s model weights and the generator model. The Generator never actually sees examples from the domain and adapt according to the Discriminator’s performance.

## 4 Results

The results of the system were analyzed in several phases. In the first phase, traditional classifiers were used to determine baseline classification accuracy. For this phase, the features used by the original authors were used. The next stage will discuss the performance of the deep learning classifier with and without data augmentation.

### 4.1 Performance Parameters

The results of the study are divided into two sections. The first section will focus on the baseline classifiers that were used to evaluate the data set. The results from these classifiers were used to determine some parameters for the deep learning classifiers. The second section will present the proposed deep neural network’s performance and its comparison with the other classifiers.

The area under Receiver-Operator Characteristic (AUROC) is an essential metric for the classifier’s ability to distinguish between the classes accurately. An AUROC value over 0.9 is considered an excellent classifier, while over 0.8 can be regarded as a good classifier. In this study, the testing indicators used were classification accuracy and the area under the curve. The definitions of the above indicators are as follows:

$$Accuracy=\frac{TP+TN}{TP+FP+TN+FN}$$ (5)

TP is the number of true positives, FN is the number of false negatives, FP is the number of false positives, and TN is the number of true negatives.

### 4.2 Performance of Baseline Models

In the original publication, a linear SVM-based classifier was implemented to calculate classification accuracies. Leave-one-out cross-validation (LOOCV) was applied to validate the dataset (Bak et al., 2019). The grand averages of binary classification accuracies were estimated at 83.4, 77.4, and 80.6% for RFT vs. LFT, RFT vs. FT, and LFT vs. FT, respectively in the original publication. The analysis revealed that RFT vs. LFT classification accuracy, significantly higher than RFT vs. FT classification accuracy. The grand average of the ternary classification accuracy was estimated at 70.4%. In the preliminary study, 27 out of 30 volunteers exceeded the theoretical chance level of ternary classification of 42.7%. In addition to the original classification method for this study, additional traditional machine learning methods were also used for comparison. The accuracy comparison of these classifiers is given in Table 1. Apart from the original authors’ SVM-based method, other methods such as Logistic Regression, Random Forest, and XGBoost were used for comparison. Logistic Regression had the worst performance while SVM performed the best. None of the traditional classifiers which were used exceeded the classification accuracy of the original authors.

**TABLE 1:** Comparison between the performance of traditional classifiers.

Model	Ternary classification accuracy%	Average AUROC
Logistic regression	58.6	0.51
Random forest	64.8	0.59
SVM	70.6	0.72
XGBoost	65.4	0.60

### 4.3 Performance of Deep Learning Models

Two deep learning models were used, one for classification and the other for data augmentation. The traditional performance metrics are compared with the stand-alone deep learning network and data augmentation network in the first phase. In the second phase, further analysis was done to determine the data generated through the data augmentation process. The deep learning classifier proposed in this study is CNN-based, which is trained using the CGAN network’s data. The CNN classifier, which was trained using only real data, obtained an accuracy of 80%.

**FIGURE 6:**
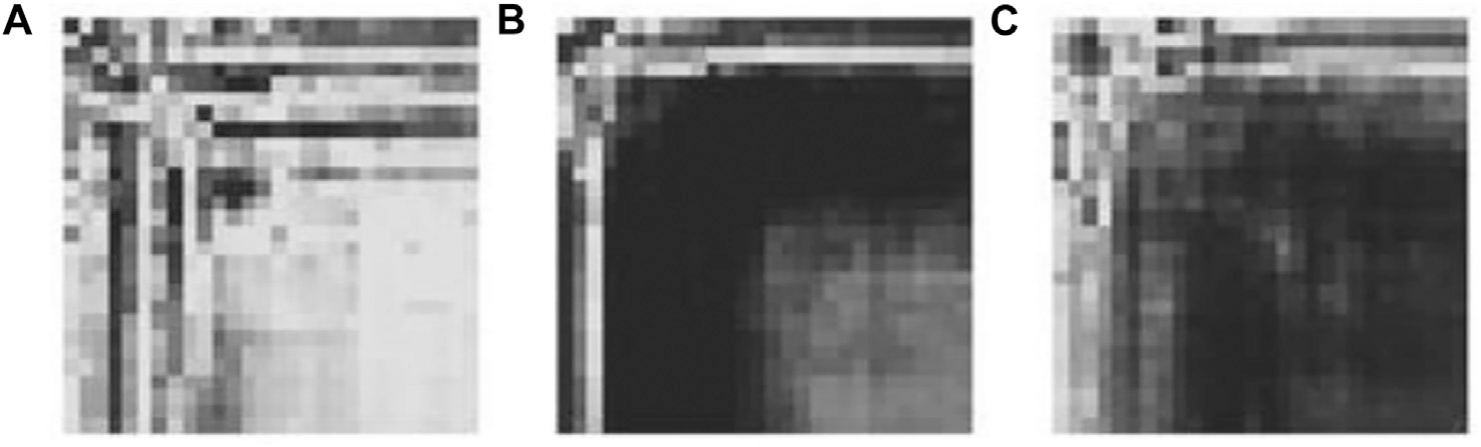
Synthetic images generated by CGAN for the tasks **(A)** Foot Tap **(B)** Left Finger Tap **(C)** Right Finger Tap.

**FIGURE 7:**
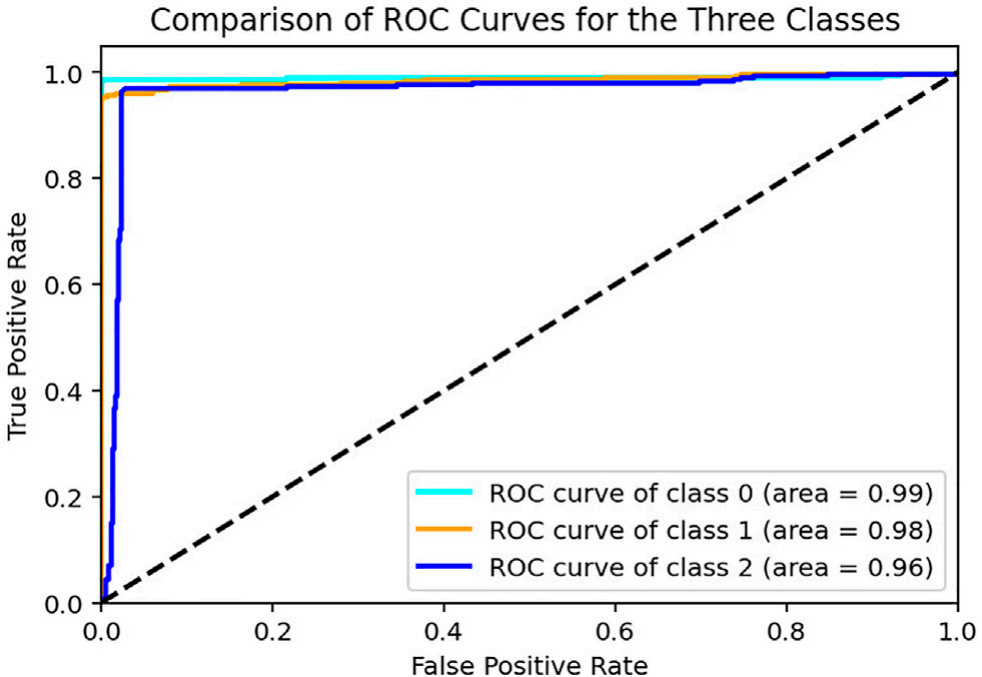
ROC curves for the three tasks obtained using all the test sets when model is trained with Real Data + 110% generated data for training set.

The sample data generated by the Generator for each category are illustrated in Figure 6. The generated data samples start at 10% of the original data and increases by 10% each step. The traditional performance metrics, such as accuracy and AUROC, and the classification accuracy are calculated for each step. Table 2 shows how the classification accuracy and average AUROC vary with each step. Further Table 3 shows the precision obtained for each class in each dataset. The confusion matrix for the classifier trained using only original data is given in Table 4. According to the confusion matrix, there were several misclassifications, primarily for RHT and LHT tasks. The confusion matrix for the classifier trained using maximum data augmentation is given in Table 5. In this confusion matrix, there is only a single misclassification. The classifier trained using the maximum augmented data obtained the best AUROC value as well. The AUROC curves obtained for this instance are shown in Figure 7. As expected, the classification accuracy improved with more data. The general architecture of the classifier was not changed. However, several regularization parameters were changed along with the increase of data. Initially, there was a strict regularization scheme due to the small size of data, and periodically the regularization was relaxed to prevent under-fitting.

**TABLE 2:** Generated data and performance metrics.

Amount of data	Ternary classification accuracy% (%)	Average AUROC
Original data set	80.00	0.79
Real data + 10% generated data	80.00	0.83
Real data + 20% generated data	83.33	0.875
Real data + 30% generated data	83.33	0.87
Real data + 40% generated data	83.33	0.91
Real data + 50% generated data	86.67	0.9
Real data + 60% generated data	90	0.92
Real data + 70% generated data	86.67	0.92
Real data + 80% generated data	90	0.94
Real data + 90% generated data	90	0.95
Real data + 100% generated data	93.33	0.97
Real data + 110% generated data	96.67	0.98

**TABLE 3:** Precision for each Class in each Data set.

Amount of data	Precision		
	LHT	RHT	FT
Original data set	0.73	0.89	0.80
Real data + 10% generated data	0.80	0.80	0.80
Real data + 20% generated data	0.91	0.80	0.78
Real data + 30% generated data	0.90	0.80	0.80
Real data + 40% generated data	0.91	0.82	0.88
Real data + 50% generated data	0.91	0.89	0.80
Real data + 60% generated data	0.91	0.90	0.89
Real data + 70% generated data	0.80	0.91	0.89
Real data + 80% generated data	0.80	0.91	1.0
Real data + 90% generated data	0.78	0.91	1.0
Real data + 100% generated data	0.89	1.0	0.91
Real data + 110% generated data	0.9	1.0	1.0

**TABLE 4:** The Confusion Matrix obtained by CNN model using test data from subject 1 for original data set.

Actual	Predicted		
	RHT	LHT	FT
RHT	8	1	1
LHT	2	7	1
FT	0	1	9

**TABLE 5:** Confusion Matrix for model with GAN data augmentation (Real Data + 110% Generated Data) using test data from subject 1

Actual	Predicted		
	RHT	LHT	FT
RHT	9	1	0
LHT	0	10	0
FT	0	0	10

The classifier obtained maximum classification accuracy of 96.67% which was trained with real data and 110% of generated data. Further improvement of the classification accuracy required the data changes to the data architecture. Since the authors intended to improve the model’s performance through data augmentation only, the data generation step concluded after 110% data. This dip in the classification accuracy after 110% may be caused by over-fitting, and further regularization may be required.

### 4.4 Performance Metrics Related to the Generative Adversarial Networks (GAN)

The conditional GAN used to classify the tasks can generate artificial samples belonging to a specific category specified by the conditional argument. This section analyzes the similarity between the generated samples and the original data distribution this section.

Different models of GANs have used other performance evaluation metrics, including inception score, mean opinion score (MOS), Wasserstein metric, fuzzy combinatorial analysis (FCA) score, log-likelihood, and human evaluation schemes (Sharma et al., 2018). Among these, the Inception score is the most popular metric (Salimans et al., 2016). However, it is insensitive to prior distribution labels. Frechet Inception distance is also sensitive to generated samples’ visual quality and more robust to noise one step further. It is capable of detecting intra-class mode dropping. Another variant is the kernel inception score. Multi-scale structural similarity for image quality (MS-SSIM) is used to interpret the diversity of images, where a higher MS-SSIM score indicates a higher similarity between the two images (Sharma et al., 2018; Wang et al., 2003; Brooks et al., 2008).

Figure 6 shows a sample of generated GASF images for the three tasks. Visual inspection was done to determine the quality of the images generated. The outputs for various inputs were inspected to see whether there is diversity among the generated images. In case of a mode collapse, the generated images will look very similar regardless of the inputs. In cases with visually similar images, MS-SIM values were used to determine how similar they are. For the proposed system, measures were taken at the generator architecture design stage, introducing Batch Normalization after each layer.

## 5 Discussion

Our study results show that NIRS based classification accuracy can be increased by using deep learning methods. Classification processes are an essential step to enhance NIRS based classification systems used in BCI applications. The proposed ternary classification system can classify RHT, LHT, and FT brain activation patterns. Some form of preprocessing was required for the classification. However, the feature selection step can be eliminated by using deep neural networks. As expected by using the GAN network for data augmentation increased the classification accuracy of the system. The proposed network can generalize the model used in the task classification of a wide range of subjects.

One drawback regarding deep neural networks is that a substantial amount of data must be used to train the network correctly. Data augmentation methods can increase the sample size when collecting many samples is not practical due to economic or time constraints. Traditional data augmentation methods include zooming, cropping and rotating, etc. Although these methods are very successful in object classification, there is no single object to focus on classification in exceptional cases like time series data represented in images. Hence, using traditional data augmentation methods makes little sense. Therefore, to improve the classification accuracy using the deep neural network’s ability to handle complex data, the authors propose a combined GAN and CNN classifier. The GAN networks have the potential to improve the performance of the classification by data augmentation. The generator-discriminator model of GAN networks enables the generation of artificial samples. This approach also introduces the unique challenge of training the model since optimization is a zero-sum game.

According to the results, it is clear that the CGAN-CNN network shows superior accuracy compared to the other machine learning methods use. Hence it is possible to train a reliable deep learning classification model with small sample sizes. Further, the authors tried to train the CNN classifier entirely with GAN-generated data and test it on real data. However, this approach was not very successful, with an average accuracy of 63.33%. For comparison, the classifier trained with the same size training set from the original data set, when evaluated with the test set, obtained an accuracy of 80%. Generally, deep learning models tend to over-fit data when they are trained using small sample sizes. This over-fitting results in poor performance with test data, causing the model’s generalization to a significant challenge. Further, it should be added that although the performance improved for the models, with data augmentation, the regularization parameters of the models changed to make sure the model’s performance does not deteriorate.

One of the most important aspects of deep learning-based models is handling the raw data without much preprocessing. By using raw data, more complex deep learning architectures may be required for classification tasks. The authors initially tried to develop a model based on images derived using raw data in this study. The raw data-based classifier performed poorly compared to the model with preprocessing. A certain degree of preprocessing was required for the deep learning models in this study. This result does not mean that future studies should abandon raw data-based classifiers. The raw data-based classifiers will be helpful if real-time task classification is needed. Hence, more research should is required to determine the correct balance between the classification accuracy and preprocessing complexity.

A further enhancement to fNIRS-based studies that the authors suggest is artificial sequence generation regarding neural signals using generative networks. The current deep learning-based generative networks are mostly focused on image studies. There may be some information lost by representing the sequences as images. Artificial neural signals can be used in studies for both brain-computer interfaces and health fields as well. Further preprocessing signals can train such generative neural networks, eliminating the preprocessing step for the additional data. Generative networks can be used to overcome the economic and time constraints of the population studies. Generative networks such as CGAN can be used along with the other neuroimaging techniques, especially where a single trial can be costly, time-consuming, or repetitive trials harmful to the subject.

## 6 Conclusion

fNIRS is a neuro-imaging technique that can be used for task classification for Brain-Computer Interfaces. The complex nature of fNIRS signals makes it ideal for deep learning-based classifiers. The small sample size gained from the experiments using neuro-imaging techniques makes it difficult for the deep learning classifiers to generalize. Generative Models can generate synthetic samples that can increase the classification accuracy of deep learning-based classifiers. The authors proposed a Conditional GAN network that enables data augmentation by generating new samples. The newly generated samples are used to train the deep learning model, which is CNN-based. Furthermore, it was observed that the classification accuracy on the test set could improve by using generated data samples for training. This proposed solution can be used in various instances where a large amount of data is required for accurate predictions from a model. Yet, due to multiple constraints, data collection is complex. The quality of the system can improve with more data collection and fine-tuning of the model. Further analysis can compare how the synthetic data generated from the GAN network with different subjects. Such synthetic neural data can be used for training both task classification and medical data classification where data collection has constraints.

## Data Availability

The original contributions presented in the study are included in the article/Supplementary Material, further inquiries can be directed to the corresponding author.
